# Predicting the achievement emotions of elementary and middle school students in online learning based on control-value theory

**DOI:** 10.3389/fpsyg.2025.1601052

**Published:** 2025-07-04

**Authors:** Yan Yi, Lixiang Gao, Shuang Wang, Mingzhang Zuo, Heng Luo

**Affiliations:** ^1^Faculty of Artificial Intelligence in Education, Central China Normal University, Wuhan, China; ^2^Munich Center of the Learning Sciences, Ludwig-Maximilians-University of Munich, Munich, Germany

**Keywords:** online learning, achievement emotions, learning experience, control-value theory, elementary and middle school students

## Abstract

**Introduction:**

Achievement emotions have a profound impact on students' academic achievements and learning strategies, especially in online learning context where students may face great challenges in maintaining positive emotions due to physical separation and social isolation. While most studies focus on achievement emotions as predictors of academic performance, fewer have examined them as outcome variables. However, achievement emotions themselves can be seen as an important learning outcome, as experiencing positive achievement emotions is also a valuable learning goal.

**Methods:**

This study explored key factors influencing achievement emotions among Chinese elementary and middle school students in online mathematics learning, using control-value theory as a framework. Data were collected using stratified sampling, and backward regressions were used to analyze data from 2,940 students. In addition, mediation analyses were conducted to examine whether control and value appraisals mediated the relationship between technology efficacy and achievement emotions.

**Results:**

Results indicated that control and value appraisals were the strongest predictors of achievement emotions, positively predicting positive emotions and negatively predicting negative emotions. Notably, negative effort belief positively predicted all three negative emotions substantially, surpassing the predictive effects of control appraisal. This study also found that technology efficacy positively predicted all positive emotions and negatively predicted anxiety. Regarding demographic factors, grade level and school tier significantly influenced some achievement emotions, but gender was not a significant predictor. In addition, mediation analyses showed that control and value appraisals partially mediated the effects of technology efficacy on both positive and negative achievement emotions.

**Conclusion:**

This study emphasizes the need to optimize online learning by enhancing students' control and value appraisals, addressing negative effort beliefs. Special attention should be given to students in disadvantaged schools and boys in all schools during online mathematics learning.

## 1 Introduction

The rapid advances in information technology, as well as the nationwide home study initiatives during the COVID-19 pandemic, have expanded online and hybrid learning to the younger student population in elementary and middle schools, presenting opportunities for nationwide online learning initiatives (Oliveira et al., 2021; Guzzo et al., 2023). However, the online transition has also introduced challenges, such as self-regulation difficulties, social isolation, and technical glitches, which necessitated enhanced learning skills like emotion regulation and time management (Kirmizi, 2015; Zuo et al., 2020, 2022). Given their limited exposure to online learning, elementary and middle school students frequently experience negative achievement emotions and burnout. This highlights the need for focused research on their unique learning conditions (Xu et al., 2013; Humphry and Hampden-Thompson, 2019).

Achievement emotions have a crucial influence on students' motivation, goal setting, the use of learning strategies, academic achievement, and wellbeing (Artino, 2012; Noteborn et al., 2012; Camacho-Morles et al., 2021; Wu and Yu, 2022). Positive emotions, such as enjoyment and pride, can foster motivation and encourage the adoption of adaptive learning strategies (Artino, 2012). For instance, Carmona–Halty et al. (2019) found that positive emotions, such as hopefulness, help students align their goals with intrinsic motivation, thereby enhancing perseverance and increasing the likelihood of higher academic performance. Conversely, negative emotions, can heighten metacognitive activity but diminish course satisfaction and motivation to persist in learning (Noteborn et al., 2012). Beyond their impact on academic outcomes, students' emotional experiences are also an important learning outcome and constitute an important factor in their mental health (Frenzel et al., 2007).

Despite the acknowledged impact of achievement emotions, research on their development in online learning environments, especially among elementary and middle school students, remains sparse (Artino, 2012; Marchand and Gutierrez, 2012). Importantly, elementary and middle school students are at a critical phase of their emotional development and are more prone to specific challenges in online learning, such as distraction and social isolation, which highlights the importance of a thorough investigation into the achievement emotions of younger learners in online settings (Artino and Jones, 2012).

The control-value theory provides a solid framework for analyzing achievement emotions in online learning (Daniels and Stupnisky, 2012; Loderer et al., 2020). It highlights the impact of students' perceived control and the value of academic achievements on their emotions (Pekrun, 2006), which is particularly relevant for online settings where these perceptions are greatly influenced by students' autonomy, technical skills, and self-efficacy. This framework also captures both positive and negative emotions holistically, thus providing a complete picture of students' emotional experiences when studying online (Daniels and Stupnisky, 2012). However, research in face-to-face environments has also emphasized several other factors that can shape students' achievement emotions, including achievement goals, effort beliefs, technology efficacy, and demographic variables such as gender and grade level. For example, achievement goals influence how students interpret academic tasks and evaluate their progress, impacting their emotional experiences. Effort beliefs, which refer to students' perceptions of whether intelligence can be developed through hard work, also play a critical role in shaping motivation and emotional responses (Dweck, 1999; Yan et al., 2014). Furthermore, technology efficacy in online learning and demographic factors like gender, grade level, and school tier have been shown to influence students' learning experiences and emotional outcomes in face-to-face learning contexts (Voyer and Voyer, 2014; Pekrun et al., 2017).

Consequently, this study incorporates psychological, technological, and demographic factors alongside the control-value framework to provide a more comprehensive understanding of the factors that contribute to both positive and negative achievement emotions among elementary and middle school students in online learning. This integrated perspective highlights how these diverse influences converge to shape students' achievement emotions, offering insights into the creation of supportive and emotionally engaging online learning environments. More specifically, the following three research questions have guided our investigation:

RQ1: what are the levels and patterns of achievement emotions (positive and negative) experienced by elementary and middle school students in online learning environments?
RQ2: what psychological, technological, and demographic factors predict positive achievement emotions (e.g., enjoyment, hopefulness, pride) among elementary and middle school students in online learning?
RQ3: what psychological, technological, and demographic factors predict negative achievement emotions (e.g., anxiety, hopelessness, boredom) among elementary and middle school students in online learning?

## 2 Literature review

### 2.1 Achievement emotions

Achievement emotions, also known as academic emotions, are emotions that are directly related to learning activities and their corresponding outcomes (Pekrun, 2017; D'Mello et al., 2024). They reflect individuals' affective responses to their academic progress, achievements, and the challenges they encounter throughout the learning process (Camacho-Morles et al., 2021). Within achievement emotions, activity emotions refer to those emotions that individuals experience during learning activities such as joy or frustration. In contrast, outcome emotions are related to learning results and are categorized as prospective or retrospective. Prospective emotions are tied to anticipated outcomes, like hope for success or fear of failure, while retrospective emotions reflect responses to achieved outcomes, such as pride after success or disappointment after failure. These outcome emotions capture students' emotional reactions to their academic achievements or setbacks. Achievement emotions include feelings such as enjoyment, pride, hopefulness, hopelessness, boredom, anxiety, shame, and anger (Pekrun et al., 2011). These emotions can be further classified by valence (positive or negative), activation (activating or deactivating), and object focus (activities or outcomes; Moreira et al., 2019). Although emotional responses show similarities across genders, ages, and grades, they are primarily shaped by students' subjective evaluation of learning events, which indicates that achievement emotions are context-specific and vary across subjects and environments (Pekrun, 2006; Moreira et al., 2019; Schukajlow et al., 2023).

### 2.2 Control-value theory and achievement emotions

Control-value theory posits that students' achievement emotions stem from their control and value appraisals of learning events (Pekrun, 2006; Yu et al., 2022). Control appraisal involves perceptions that learning activities or outcomes are controllable, while value appraisal relates to the significance that learners attribute to these activities and outcomes (Pekrun, 2006). Positive emotions tend to emerge when learners perceive high control and value, while negative emotions arise when these perceptions are lacking. Most evidence supporting this theory comes from face-to-face settings (Frenzel et al., 2007; Marchand and Gutierrez, 2012; Noteborn et al., 2012). However, recent studies have extended the application of control-value theory to online learning environments suggesting that its core mechanisms remain applicable even though online contexts differ by offering more learner autonomy and less direct interaction (Guetzoian, 2022). For example, Yang et al. (2021) found that technical challenges and teacher support in online English classes shaped students' enjoyment and anxiety through their impact on control and value appraisals. Similarly, Kaiqi and Kutuk (2024) demonstrated that clear instructional structures and teachers' digital competence enhanced online students' control and value appraisals, fostering positive emotions. Building on these findings, we hypothesize that in online learning contexts, students' control and value appraisals positively predict positive emotions and negatively predict negative emotions.

Additionally, technology efficacy may impact students' control and value appraisals, as their perceptions of how effective digital tools are can influence their sense of competence and the value they attach to learning activities. Research has shown that higher technology efficacy is linked to more positive control and value appraisals, which in turn affect students' achievement emotions (Stilin et al., 2024). Therefore, we hypothesize that control and value appraisals mediate the relationship between technology efficacy and achievement emotions in online learning contexts.

### 2.3 Achievement goals and achievement emotions

Based on conceptual corollaries and extensions of control-value theory, achievement goals are commonly understood as reasons for engaging in achievement-oriented behaviors, influencing a wide range of cognitive and emotional responses in students (Elliot, 1999; Bong, 2001; Pekrun, 2006). Elliott and Dweck (1988) initially classified achievement goals into two types: performance and learning goals. The performance goal focuses on demonstrating ability and avoiding negative evaluations, with individuals striving to validate their competence to others. However, the learning goal emphasizes personal growth and mastery, where individuals seek to enhance their abilities over time. Elliot and McGregor (2001) renamed them performance and mastery goals and further subdivided them. In this study, we use outcome and ability goals to refer to both constructs. An outcome goal focuses on final academic performance, may elicit emotions like hope and pride for positive outcomes, and anxiety or hopelessness for negative outcomes (Hu et al., 2024). On the other hand, an ability goal, which focuses on developing competence and acquiring knowledge, aligns with the concept of task orientation as described by Duda and Nicholls (1992). Their research found that such goals promote activity-related enjoyment and reduce boredom, as individuals derive intrinsic satisfaction from learning and personal growth, rather than relying on external approval. Therefore, we hypothesize that ability goals may be stronger predictors of positive emotions compared to outcome goals.

### 2.4 Effort beliefs and achievement emotions

Effort beliefs refer to individuals' beliefs about whether intelligence can be improved through hard work (Dweck, 1999; Yan et al., 2014). Within the framework of control-value theory, effort beliefs are considered part of the expectation component, which is inherently linked to both control and value appraisals (Pekrun, 2006). When students believe that intelligence can be developed through effort, they are more likely to see learning activities as valuable, which in turn may increase their motivation and positive emotional experiences. This positive view can help sustain effort and engagement, while negative effort beliefs might lead to emotional disengagement and reduced effort (Lam et al., 2008). Research in online learning settings has also provided evidence for these associations. For instance, Ebn-Abbasi et al. (2024) found that in online English courses at Iranian private language institutes, students with growth-oriented effort beliefs reported more enjoyment and less anxiety. Similarly, Yang et al. (2024) observed that positive effort beliefs in online English courses were associated with higher positive emotions and fewer negative emotions. In a related line of research, Shen et al. (2024) showed that students with incremental views of intelligence, which emphasize the belief that ability can be developed through effort, demonstrated higher levels of autonomous motivation and engagement in online learning. Based on these findings, this study hypothesizes that positive effort beliefs will positively predict positive emotions and negatively predict negative emotions among elementary and middle school students in online learning environments.

### 2.5 Online learning technology efficacy and achievement emotions

Online learning technology efficacy reflects students' confidence in their ability to utilize online tools and resources to learn in an online environment. According to Bandura's self-efficacy theory, this confidence may influence their perceived control and serve as a catalyst for positive achievement emotions (Bandura, 1997), as students are more likely to experience positive achievement emotions when they believe that they can effectively use online learning technologies (Ahmed et al., 2010; An et al., 2022). However, technical difficulties encountered in online learning can lead to negative achievement emotions such as anxiety, frustration, and boredom (Artino and Jones, 2012). In addition, technology efficacy is related to students' ability to adapt to the online learning environment (Awee et al., 2022). Students with high technology efficacy are more likely to make a smooth transition to this new mode of learning because they believe they can overcome technological barriers and effectively use online platforms for self-directed learning and communication. This adaptability not only promotes learning efficiency, but also helps them connect with their peers and receive needed social support, both of which are important for students to develop positive achievement emotions (Forsblom et al., 2021; Lee et al., 2021). Considering these findings, we hypothesize that higher technology efficacy in online learning will increase the likelihood of students experiencing positive emotions, whereas lower technology efficacy may contribute to negative emotions.

### 2.6 Demographic characteristics and achievement emotions

Demographic characteristics such as gender, grade, and school tier are possible factors that influence students' achievement emotions. Due to differences in gender roles and norms, males and females may differ in the expression and experience of achievement emotions, and these differences may be shaped by socialization processes and cultural contexts. Research has shown that girls tend to report higher levels of negative emotions (e.g., anxiety, boredom, shame) than boys (Bender et al., 2012; Pekrun et al., 2017). Grade level, on the other hand, serves as a predictor variable reflecting the different academic pressures and expectations that students may face at different stages of learning. As grade level increases, academic difficulty and competitive pressure may increase (Gao et al., 2022; Meyer and Schlesier, 2022), which may affect students' achievement emotions. School tiers represent the different educational resources and learning environments that students are exposed to. The quality of instruction, faculty, educational resources, and school culture of schools in different tiers may have an imperceptible impact on students' achievement emotions (Frenzel et al., 2007; Tempelaar et al., 2012). Based on these findings, we hypothesize that in online learning environments, female students will experience more negative emotions than male students. Additionally, with increasing grade level, students will experience more negative and fewer positive emotions. Finally, students in higher school tiers will experience more positive emotions than those in lower school tiers.

### 2.7 A summary of possible influencing factors of achievement emotions

In sum, our literature review has identified 10 factors that possibly influence students' online learning achievement emotions. Gender, grade, and school tier are included because they are key demographic variables. Control appraisal, value appraisal, outcome goal, ability goal, positive effort belief and negative effort belief are considered key predictors because they are important constructs drawn from control-value theory. Lastly, technology efficacy is included because, in the context of online learning, students' technology efficacy may have a significant impact on their achievement emotions.

Moreover, this study focused on six emotions: enjoyment, hopefulness, pride, anxiety, hopelessness and boredom. The exclusion of shame and anger was based on two main considerations. First, during the COVID-19 pandemic, our research team conducted shadow observations and interviews in classrooms in Hubei province. According to our qualitative data, these emotions were rarely observed. This suggests that these emotions may not be as prevalent in the context of online learning for elementary and middle school students. Second, both shame and anger differ from the selected emotions in its attribution mechanisms and stability. These emotions are fleeting and event-induced emotions, rather than stable emotional states. For example, anger can be triggered by perceptions of external injustice (Mameli et al., 2022), such as when a student feels unfairly treated. Since these emotions are often triggered by specific events, they tend to fluctuate quickly, which makes them difficult to measure consistently. For these reasons, shame and anger were not included in this study.

### 2.8 Literature gap and purpose of the present study

**Figure 1:**
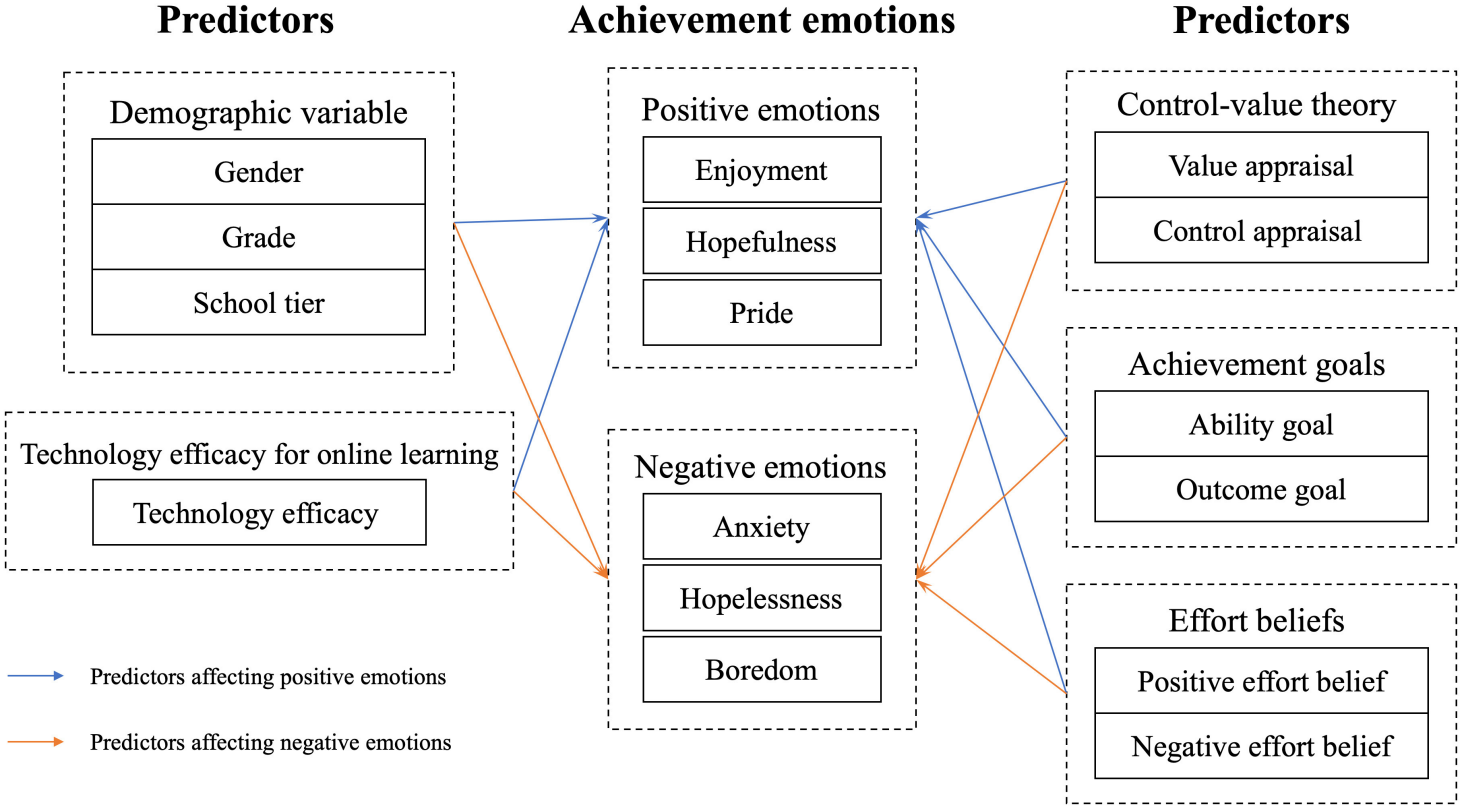
Achievement emotions and potential predictors.

While much research has investigated the relationship between achievement emotions and academic outcomes (Lin and Yin, 2024; Wang et al., 2024), most studies have primarily treated achievement emotions as predictor variables, with limited attention given to their role as outcome variables. This gap is particularly notable among elementary and middle school students, a group that is undergoing critical emotional development. Achievement emotions are important at this stage, influencing motivation, goal setting, learning strategies, academic achievement, and wellbeing. To address this literature gap, this study innovatively examined achievement emotions as outcome variables and systematically explores their influencing factors in the context of online mathematics learning, extending the application of control-value theory to this unique educational setting. Figure 1 presents the conceptual framework of the study, outlining the key variables examined, including students' achievement emotions and their potential antecedents. Furthermore, the shift to emergency remote teaching due to the pandemic also provided a valuable opportunity to study the achievement emotions of elementary and middle school students in the online learning context for an extended period of time.

## 3 Method

### 3.1 Ethical statement

The research protocol for this study received an exemption from the Central China Normal University Institutional Review Board (CCNU IRB). The survey was conducted online without direct contact with participants, and strict measures were implemented to ensure the confidentiality of personally identifiable information. All data was coded in a manner that prevents linking back to individual participants, ensuring privacy and data security throughout the research process. Before completing the questionnaire, participants were informed about the objectives of the study. Participation was entirely voluntary, with individuals having the right to withdraw at any time or to skip specific questions if they chose to do so.

### 3.2 Research context and participants

Since February 2020, elementary and middle schools across the country have adopted online teaching methods to organize students' learning in response to the COVID-19 pandemic. The methodology used in this study involved the collection and analysis of electronic questionnaires administered to participants after one and a half months of online learning. The data were collected between March 18 and March 27, 2020. We selected participants from grades 4 to 9 in eight different schools. This age range ensures participants have the cognitive abilities to understand and respond accurately to our surveys. It also aligns with educational policies of the government, which does not mandate online learning for younger students in grades 1–3, given their developmental needs, particularly in terms of health and vision. At the same time, we have selected eight schools in different geographical locations in Hubei province, Guangdong province and Chongqing municipality. Hubei province, as the region where the COVID-19 first broke out and was most severely affected, is representative of the policies and challenges facing the education system. The inclusion of Guangdong province and Chongqing municipality, as the economic and cultural centers of southern and southwestern China respectively, ensures that the study covers areas with different levels of economic development and educational resources, thus increasing the breadth and depth of the study.

In selecting the participants, we paid special attention to differences among three types of schools: disadvantaged schools, typical schools, and key schools. Disadvantaged schools are schools with relatively limited resources and facilities, usually located in rural areas. Typical schools are usually located in small urban areas. Key schools, on the other hand, typically found in metropolitan areas and offer enhanced educational experiences due to their rich resources and advanced facilities.

To collect data, school principals and class teachers were commissioned to distribute questionnaires. A total of 3,788 questionnaires were collected. After excluding invalid questionnaires, which were responses completed in < 40 s or with identical answers throughout, the final dataset comprised 2,940 valid questionnaires, with a valid response rate of 77.6%. The gender, grade, and school tier distribution of the respondents is presented in Table 1.

**Table 1:** Participants' demographic characteristics.

**Variable**	**Category**	**Sample size**	**Percentage (%)**
Gender	Male	1,509	51.33
	Female	1,431	48.67
Grade	Grade 4	490	16.67
	Grade 5	721	24.52
	Grade 6	537	18.27
	Grade 7	316	10.75
	Grade 8	437	14.86
	Grade 9	439	14.93
School tier	Disadvantaged schools	1,062	36.12
	Typical schools	945	32.14
	Key schools	933	31.73

### 3.3 Survey instrument

The instrument used in this survey was a 45-item questionnaire (see Appendix A) with three sections. The first section gathered basic demographic information such as gender and grade. The second section contains 28 items on a 5-point Likert scale to investigate factors that may affect achievement emotions, including respondents' technology efficacy (7 items), value appraisal (5 items), control appraisal (5 items), effort beliefs (5 items), and achievement goals (6 items). The third section contains information from the Achievement Emotions Questionnaire (AEQ; Pekrun et al., 2011) The present study selected six emotional dimensions in the questionnaire: enjoyment, hopefulness, pride, anxiety, hopelessness, and boredom. To avoid the lengthy questionnaire for younger students, two representative items were chosen for each emotional dimension. Each dimension includes one item measuring emotions specific to the classroom setting and another measuring emotions specific to the learning process. For example, the two topics contained in enjoyment are: (1) “I quite enjoy being in the online math class.” and (2) “I enjoy acquiring knowledge in the online math class.” The α coefficients of internal consistency for each dimension are 0.881, 0.886, 0.918, 0.891, 0.957, and 0.931.

The questionnaire for the online learning technology efficacy was based on the learner's technical use self-efficacy and online communication self-efficacy from the Online Learning Readiness Scale (OLRS). The value assessment questionnaire was adapted from the online learning value section of the Artino Online Learning Value and Self-Efficacy Scale, and the control assessment questionnaire was adapted from the Bentridge Learning Motivation Scale (MSLQ; Yilmaz, 2017). The effort beliefs questionnaire was adapted from Blackwell's instrument (Hung et al., 2010) to measure students' effort beliefs. For example, a representative item for negative effort belief is: “I believe that if I'm not intelligent, no matter how hard I study, I won't perform well.” Conversely, a sample item for positive effort belief reads: “When something is challenging, I put in more effort to complete it.” The achievement goal questionnaire was adapted from Grant et al.'s Achievement Goal Inventory Scale (Artino and McCoach, 2008). The distribution of survey items is shown in Table 2.

**Table 2:** The constructs, structure, reliability, and validity results of the questionnaire.

**Constructs**	**Items**	**Cronbach's α**	**Factor loading**	**CR**	**AVE**	**√AVE**
Technology efficacy	6–12	0.860	[0.432–0.827]	0.893	0.551	0.742
Value appraisal	13–17	0.898	[0.716–0.857]	0.901	0.645	0.803
Control appraisal	18–22	0.851	[0.494–0.855]	0.869	0.577	0.760
Negative effort belief	23–24	0.727	[0.663–0.865]	0.742	0.594	0.771
Positive effort belief	25–27	0.786	[0.729–0.752]	0.787	0.552	0.743
Achievement goals: outcome	28–30	0.686	[0.630–0.766]	0.727	0.472	0.687
Achievement goals: ability	31–33	0.828	[0.768–0.823]	0.832	0.623	0.789

A questionnaire's construct validity is determined by both its convergent and its discriminant validity (Campbell and Fiske, 1959). Convergent validity is the degree of the indicators' shared variance in measuring a potential construct and can be calculated through the standardized factor loadings of the items, composite reliability (CR), and average variance extracted (AVE). According to Bagozzi and Yi (1988), a CR larger than 0.6 and an AVE larger than 0.5 are considered acceptable. As shown in Table 2, majority of variables in the questionnaire meet these thresholds, with only a few exceptions. Given the established nature and validated history of the scales used in the questionnaire, the questionnaire can be deemed as having suitable convergent validity.

Discriminant validity is the degree to which the measures of different constructs are unrelated. According to Fornell and Larcker (1981), discriminant validity can be proven if the square root of the AVE value of a potential construct is larger than its correlation coefficients with other constructs. As shown in Table 2, the √AVE values range from 0.687 to 0.803 for all of the questionnaire's constructs and are all larger than the relevant correlation coefficients. The full correlation matrix is provided inAppendix B for reference. Thus, the discriminant validity of the questionnaire is suitable as well.

To mitigate the potential impact of common method bias resulting from the use of self-report measures, we adopted several procedural remedies, including assurances of anonymity, careful item placement, and the use of validated multi-item scales with varied item wording and formats (Podsakoff et al., 2003, 2024). In addition, we conducted Harman's single-factor test as a *post hoc* diagnostic. Results showed that the first factor accounted for 43.79% of the total variance, which is below the accepted threshold of 50% (Podsakoff et al., 2024; Podsakoff and Organ, 1986), suggesting that common method variance was not a serious concern in this study.

### 3.4 Data analysis

Visual FoxPro 6.0 was used for data entry and management. SPSS version 17.0 was used for all statistical analyses. The study first conducted descriptive statistics on the collected questionnaire data to determine the general emotions of students participating in online learning and to deduce the characteristics of achievement emotions exhibited by elementary and middle school students. Six multiple regression models were then constructed using SPSS 17.0 to establish multiple linear regression models to explore the significant influencing factors on students' achievement emotions. The six emotions, including three positive emotions (enjoyment, hopefulness, and pride) and three negative emotions (anxiety, hopelessness, and boredom), served as outcome variables. A total of 10 predictors were included in the analyses: gender, grade, school tier, online learning technology efficacy, control appraisal, value appraisal, positive effort belief, negative effort belief, ability goal, and outcome goal. Separate regression models were run for each of the six achievement emotions as outcome variables.

To better understand the pathways through which technology efficacy influences achievement emotions, we focused on control appraisal and value appraisal as potential mediators. Based on the literature review in section 2.2, which highlighted the role of control and value appraisals in shaping achievement emotions, we conducted mediation analyses to explore how control appraisal and value appraisal mediate the relationship between technology efficacy and the six achievement emotions: enjoyment, hopefulness, pride, anxiety, hopelessness, and boredom. Specifically, we estimated 12 single-mediator models using the PROCESS macro (Hayes, 2013) with 5,000 bootstrap samples. For each of the six achievement emotions, we ran two separate mediation analyses: one with control appraisal as the mediator and another with value appraisal as the mediator.

## 4 Results

### 4.1 Descriptive statistics and correlation analysis

**Figure 2:**
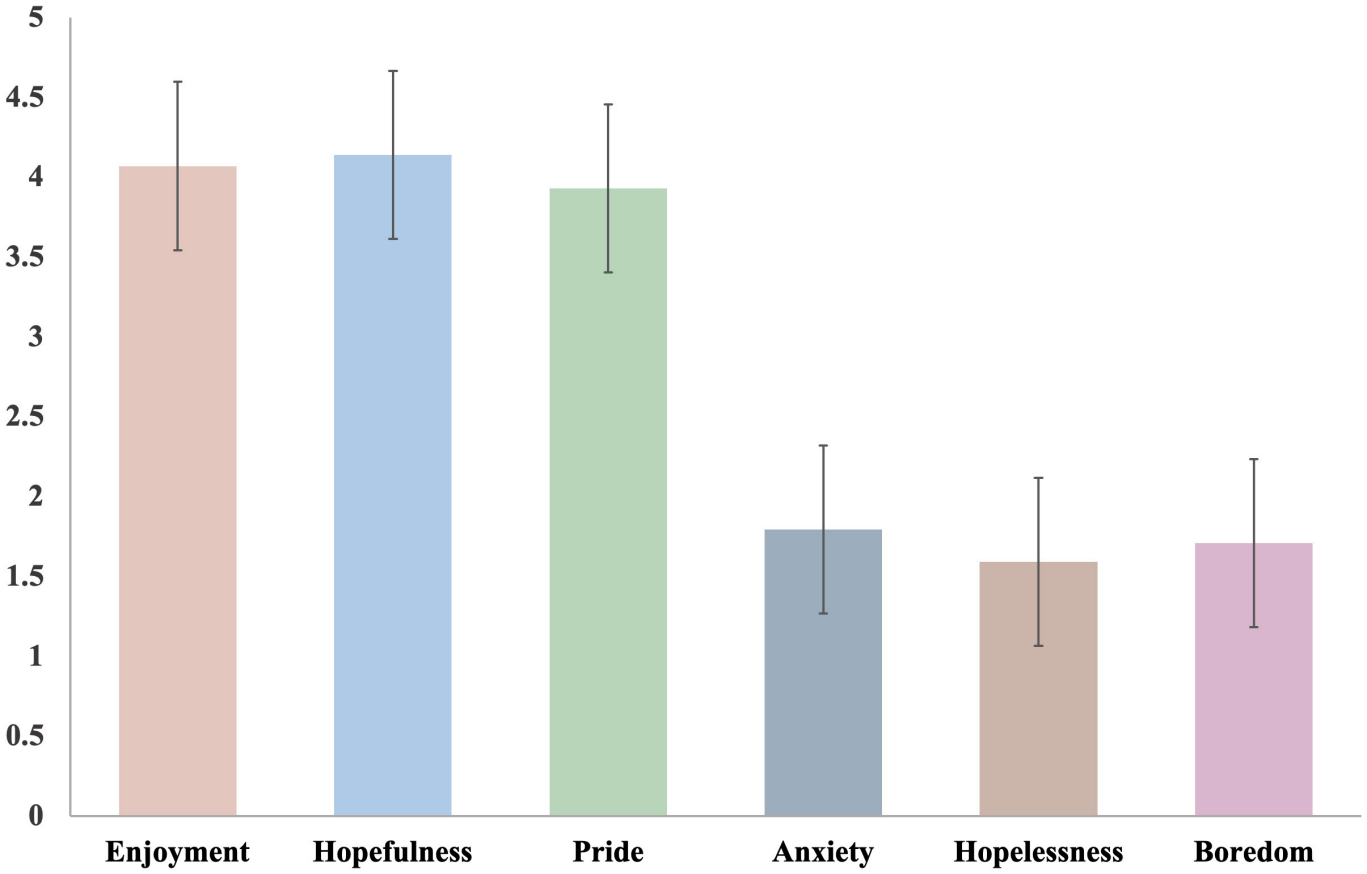
Average scores of six emotions based on five-point Likert scale ratings.

To address RQ1 on the levels and patterns of achievement emotions, we first present descriptive statistics. The mean scores of students' achievement emotions are shown in Figure 2. Positive emotions (enjoyment, hopefulness, and pride) scored higher than negative emotions (anxiety, hopelessness, and boredom). Hopefulness emerged as the most prominent positive emotion, while hopelessness had the lowest average score among negative emotions. Table 3 presents the mean scores of the six achievement emotions stratified by demographic variables, including gender, school tier, and grade. There were notable differences in the emotional experiences of different student groups, including gender differences in students' achievement emotion scores. Girls experienced more positive and fewer negative emotions than boys. Compared to students in typical and key schools, students in disadvantaged schools scored the lowest in positive emotions and the highest in negative emotions during online learning. There were also differences in the achievement emotion scores of students at different grade levels. Students' positive emotions (enjoyment, hopefulness, pride) tend to increase as grade level increases, particularly among the student population in grades 4–6. Concurrently, negative emotions (anxiety, hopelessness, boredom) decrease with grade level or remain steady at low levels.

**Table 3:** Mean achievement emotions scores by gender, school tier, and grade.

**Demographic variables**	**Enjoyment**	**Hopefulness**	**Pride**	**Anxiety**	**Hopelessness**	**Boredom**
**Gender**						
Female	4.11	4.18	3.95	1.76	1.57	1.67
Male	4.03	4.10	3.91	1.82	1.61	1.75
**School tier**						
Disadvantaged schools	3.89	3.95	3.71	2.01	1.76	1.87
Typical schools	4.19	4.29	4.09	1.64	1.45	1.59
Key schools	4.15	4.20	4.01	1.70	1.54	1.66
**Grade**						
Grade 4	4.00	4.08	3.86	1.86	1.66	1.78
Grade 5	4.10	4.18	3.99	1.67	1.54	1.65
Grade 6	4.13	4.26	4.08	1.69	1.51	1.65
Grade 7	4.05	4.07	3.86	1.94	1.65	1.76
Grade 8	4.02	4.05	3.82	1.85	1.62	1.74
Grade 9	4.08	4.13	3.88	1.88	1.63	1.72

The average score for the online learning technology efficacy was 4.17. The mean scores for the value and control appraisals were 4.25 and 3.77, respectively. In addition, the mean scores for students' outcome and ability goals were 4.26 and 4.17, respectively. The average score for students' positive effort belief was 4.16, and it was 2.11 for negative effort belief. The correlation between most predictors was in the range of 0.4–0.6 (detailed information can be found in Appendix B), and the correlation between some variables was slightly larger, exceeding 0.7, but the tolerances of all predictors were between 0.2 and 1, thus passing the collinearity diagnosis. All variables were therefore added to the regression model for regression analysis.

### 4.2 Multiple regression analysis of achievement emotions in online learning

To address RQ2 and RQ3, we used gender, grade, school tier, technology efficacy, control appraisal, value appraisal, effort beliefs, and achievement goal orientation as independent variables. The six dependent variables were achievement emotions including enjoyment, hopefulness, pride, hopelessness, anxiety, and boredom. Six regression models were obtained using backward elimination multiple linear regression. The model passes the Durbin-Watson test, indicating that there is no autocorrelation in the model.

#### 4.2.1 Positive emotions: enjoyment, hopefulness, and pride

The coefficients of determination (*R*^2^) for the three positive achievement emotion models (enjoyment, hopefulness, and pride) were 0.624, 0.683, and 0.564, respectively (see Table 4). The adjusted *R*^2^ values were 0.623, 0.683, and 0.563, respectively. The tolerances of each predictor variable in the three prediction models were mostly >1–*R*^2^, and the variance inflation factor (VIF) value of each predictor variable was within 5, which indicates multicollinearity was not an issue (Marcoulides and Raykov, 2019).

**Table 4:** Multiple linear regression analysis: three positive achievement emotions (Enjoyment, hopefulness, pride).

**Predictors**	**Enjoyment**		**Hopefulness**		**Pride**	
	**Beta**	**Tolerance**	**Beta**	**Tolerance**	**Beta**	**Tolerance**
Gender	−0.020	0.991	—	—	—	—
Grade	0.064^***^	0.943	0.043^***^	0.945	0.029^*^	0.945
School tier	0.047^***^	0.949	0.037^***^	0.946	0.055^***^	0.946
Technology efficacy	0.074^***^	0.473	**0.106** ^***^	0.471	0.095^***^	0.471
Control appraisal	**0.230** ^***^	0.427	**0.325** ^***^	0.427	**0.327** ^***^	0.427
Value appraisal	**0.509** ^***^	0.391	**0.432** ^***^	0.378	**0.271** ^***^	0.378
Ability goal	—	—	−0.028^*^	0.660	0.087^***^	0.661
Positive effort belief	0.041^**^	0.604	0.057^***^	0.557	0.078^***^	0.557
Negative effort belief	−0.035^**^	0.783	−0.057^***^	0.782	−0.052^***^	0.782
*R* ^2^	0.624		0.683		0.564	
Adjusted *R*^2^	0.623		0.683		0.563	

* *p* < 0.05,

** *p* < 0.01,

*** *p* < 0.001. Beta values >0.1 are in bold to highlight their significance.

For the model predicting positive emotions, value appraisal and control appraisal had the strongest predictive ability for enjoyment, followed by technology efficacy, grade, and positive effort belief. For hopefulness, value appraisal, control appraisal, and technology efficacy had strong predictive capabilities. Control appraisal and value appraisal also had strong predictive ability for pride, while technology efficacy, ability goal, and positive effort belief were also predictive of pride. By comparing the predictive variables for the achievement emotions from the three emotion models, it appeared that the regression coefficients for value appraisal and control appraisal had a strong predictive ability for enjoyment, hopefulness, and pride. These findings suggest that students who recognize the value of online learning may report higher levels of enjoyment, hopefulness, and pride. In the three positive emotion models, technology efficacy was retained as a significant predictor. The learner's positive effort belief was found to positively predict all three types of positive emotions, while negative effort belief showed a significant negative predictive effect on these emotions. It is noteworthy that the backward method was used in the linear regression analysis, and as a result, the outcome goal was excluded from the model due to its lack of significant predictive effect on the three positive emotions.

#### 4.2.2 Negative emotions: hopelessness, anxiety, and boredom

The coefficients of determination (*R*^2^) for the three negative emotion models (hopelessness, anxiety, and boredom) are 0.341, 0.361, and 0.407, and the adjusted *R*^2^ values are 0.340, 0.359, and 0.406, respectively (as shown in Table 5). Excluding technology efficacy, control appraisal, and value appraisal, the tolerances of all the other independent variables were >1–R^2^. However, the VIF values for technology efficacy, control appraisal, and value appraisal in the model were < 5, which indicates multicollinearity was not an issue.

**Table 5:** Multiple linear regression analysis: three negative achievement emotions (Anxiety, hopelessness, boredom).

**Predictors**	**Anxiety**		**Hopelessness**		**Boredom**	
	**Beta**	**Tolerance**	**Beta**	**Tolerance**	**Beta**	**Tolerance**
Gender	—	—	—	—	0.026	0.994
Grade	—	—	−0.039^**^	0.988	−0.049^***^	0.985
School tier	−0.080^***^	0.954	−0.059^***^	0.981	−0.037^*^	0.981
Technology efficacy	−0.065^**^	0.488	—	—	—	—
Control appraisal	**−0.111** ^***^	0.428	–0.106^***^	0.476	**−0.104** ^***^	0.476
Value appraisal	**−0.264** ^***^	0.391	–0.357^***^	0.418	**−0.409** ^***^	0.418
Outcome goal	—	—	−0.030	0.709	−0.035^*^	0.708
Positive effort belief	−0.048^**^	0.618	—	—	—	—
Negative effort belief	**0.238** ^***^	0.784	**0.247** ^***^	0.852	**0.237** ^***^	0.852
*R* ^2^	0.341		0.361		0.407	
Adjusted *R*^2^	0.340		0.359		0.406	

* *p* < 0.05,

** *p* < 0.01,

*** *p* < 0.001. Beta values >0.1 are in bold to highlight their significance.

In the anxiety model, value appraisal, negative effort belief, and control appraisal demonstrated relatively strong predictive power, with value appraisal and control appraisal linked to reduced anxiety and negative effort belief to increased anxiety. School tier and technology efficacy had smaller but significant effects on anxiety. In the hopelessness emotion model, value appraisal, negative effort belief, and control appraisal were significant predictors, with students in lower school tiers experiencing higher level of hopelessness. For boredom, value appraisal showed the strongest predictive effect, reducing boredom, while negative effort belief and control appraisal also significantly predicted boredom. Grade, school tier and outcome goal further contributed to the model, with an adjusted *R*^2^ of 0.406. Comparing the predictor variables across the three emotion models, control appraisal and value appraisal negatively predicted anxiety, hopelessness, and boredom. However, negative effort belief had a positive and strong predictive value for the three negative emotions. Control appraisal, value appraisal, and negative effort belief were the predictive variables shared by the three negative emotions. Additionally, school tier and the online learning technology efficacy may predict anxiety, and grade variables showed a negative predictive ability for hopelessness and boredom.

### 4.3 Mediation analysis

**Figure 3:**
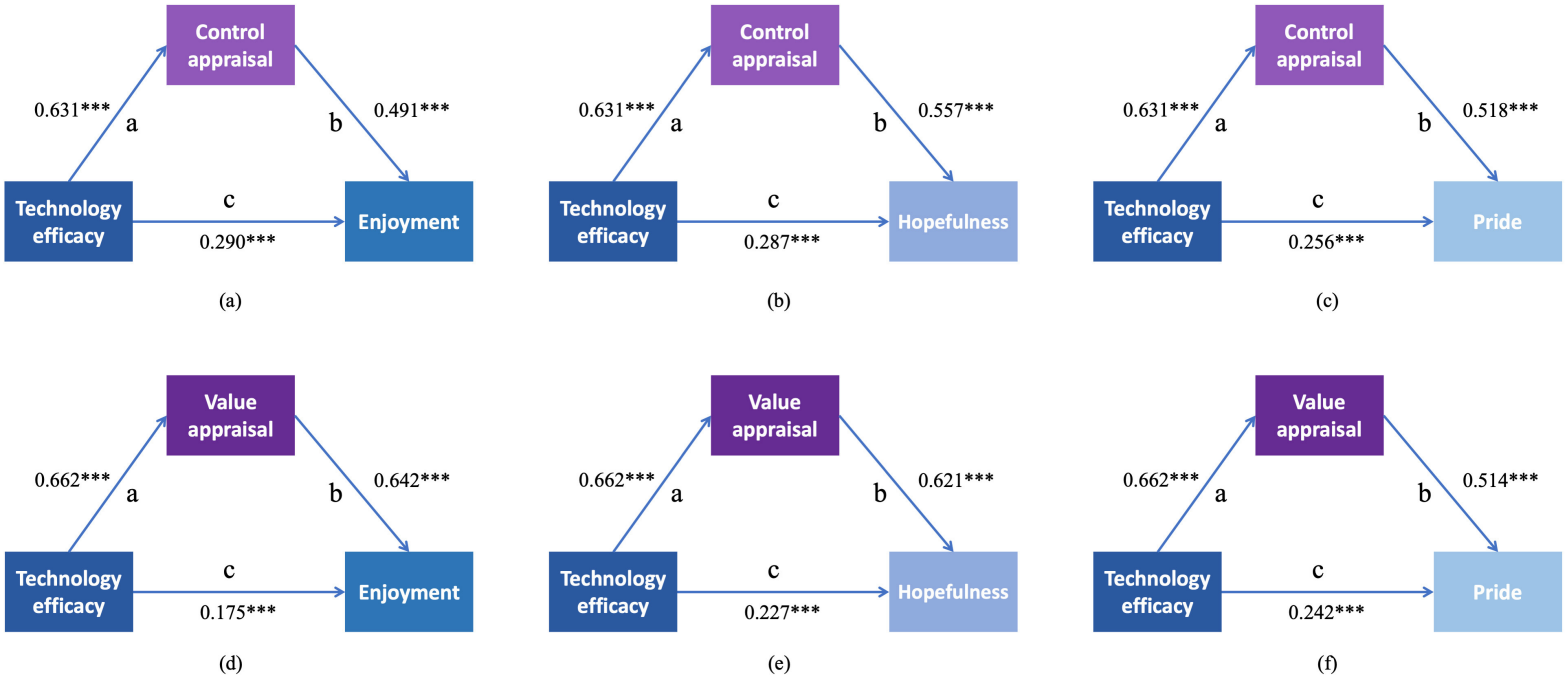
Mediation effects of control appraisal and value appraisal on the relationships between technology efficacy and positive achievement emotions (enjoyment, hopefulness, and pride). **(a)** Mediation of control appraisal between technology efficacy and enjoyment. **(b)** Mediation of control appraisal between technology efficacy and hopefulness. **(c)** Mediation of control appraisal between technology efficacy and pride. **(d)** Mediation of value appraisal between technology efficacy and enjoyment. **(e)** Mediation of value appraisal between technology efficacy and hopefulness. **(f)** Mediation of value appraisal between technology efficacy and pride. In these models, path “a” represents the effect of technology efficacy on the mediator; path “b” represents the effect of the mediator on the dependent variable (controlling for technology efficacy); path “c” represents the direct effect of technology efficacy on the dependent variable (controlling for the mediator). The indirect effect is calculated as a × b, and the total effect as c + (a × b).

**Figure 4:**
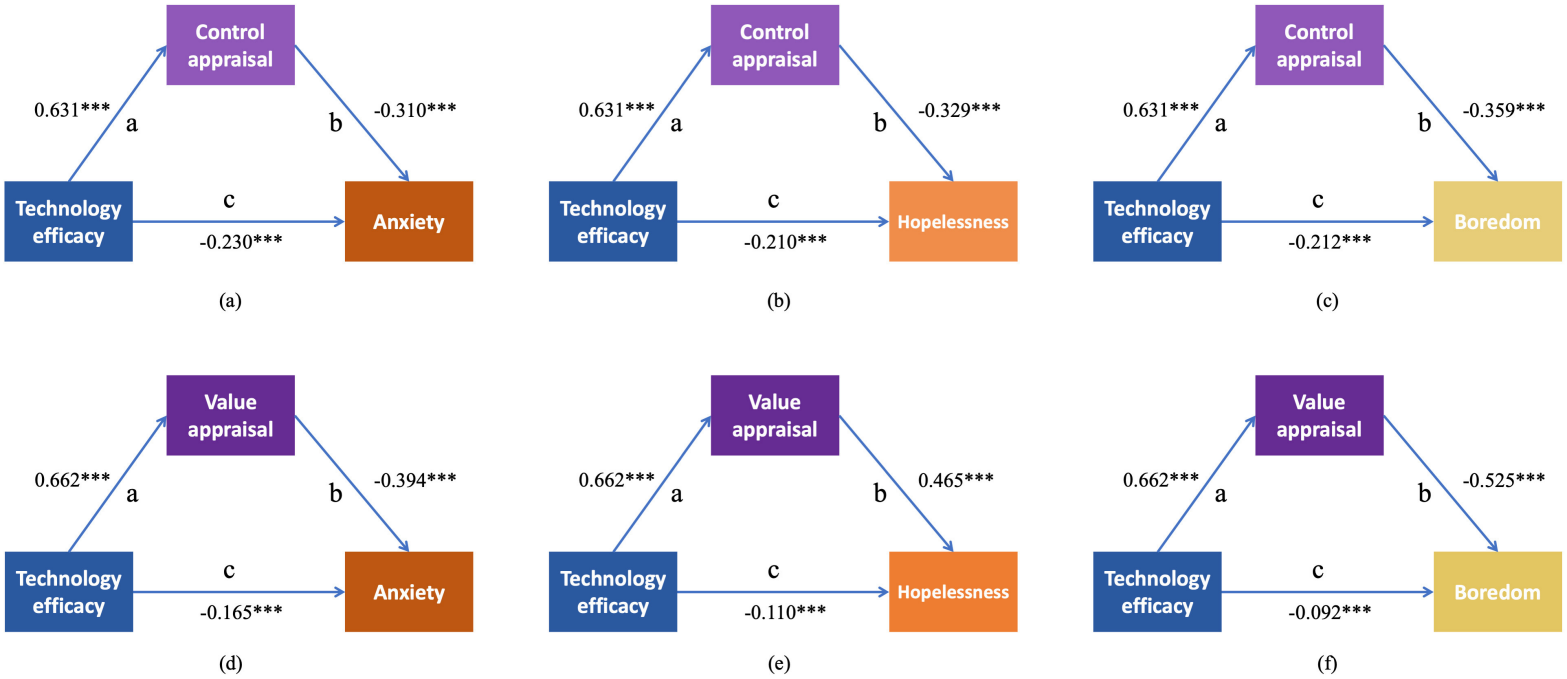
Mediation effects of control appraisal and value appraisal on the relationships between technology efficacy and negative achievement emotions (anxiety, hopelessness, and boredom). **(a)** Mediation of control appraisal between technology efficacy and anxiety. **(b)** Mediation of control appraisal between technology efficacy and hopelessness. **(c)** Mediation of control appraisal between technology efficacy and boredom. **(d)** Mediation of value appraisal between technology efficacy and anxiety. **(e)** Mediation of value appraisal between technology efficacy and hopelessness. **(f)** Mediation of value appraisal between technology efficacy and boredom. In these models, path “a” represents the effect of technology efficacy on the mediator; path “b” represents the effect of the mediator on the dependent variable (controlling for technology efficacy); path “c” represents the direct effect of technology efficacy on the dependent variable (controlling for the mediator). The indirect effect is calculated as a × b, and the total effect as c + (a × b).

The mediation analyses revealed that control appraisal and value appraisal partially mediated the effects of technology efficacy on each of the six achievement emotions. As shown in Figure 3, for positive emotions, higher technology efficacy was associated with higher levels of control appraisal and value appraisal, which were further linked to higher levels of enjoyment, hopefulness, and pride. As shown in Figure 4, for negative emotions, higher technology efficacy was similarly associated with higher levels of control appraisal and value appraisal, which were in turn associated with lower levels of anxiety, hopelessness, and boredom. Full standardized path coefficients for all single-mediator models are presented in Figures 3, 4. Detailed data for all 12 single-mediator models can be found in Appendix C.

## 5 Discussion

The results showed that control appraisal, value appraisal, and school tier align with the hypotheses regarding their predictive effects on achievement emotions. Specifically, control and value appraisals were found to enhance positive emotions while mitigating negative emotions in online learning contexts. Previous research (Shao et al., 2020) has similarly reported the positive predictive effects of control and value appraisals on positive emotions and their negative predictive effects on negative emotions in face-to-face foreign language learning classrooms. Putwain and Daumiller (2023) also observed similar results in face-to-face secondary school classrooms in the UK. Our findings extend the application of control-value theory to online mathematics classrooms, providing new insights into its relevance in digital learning environments. The findings related to school tier were also consistent with the hypotheses. A meta-analysis (Greenwald et al., 1996) highlighted a positive association between sufficient resources and student achievement. As academic achievement influences achievement emotions, the superior resources typically found in higher-tier schools likely explain why students in these schools experience stronger positive emotions and weaker negative emotions.

The findings on effort beliefs, technology efficacy, and achievement goals partially supported the hypotheses. Positive effort belief predicted all positive emotions and reduced anxiety, while negative effort belief had the opposite effect, significantly increasing negative emotions. These results echo Inzlicht et al.'s (2018) view of effort as both aversive and rewarding, suggesting that positive effort beliefs may buffer the negative emotional costs of effort and enhance the positive achievement emotions. Besides, consistent with prior studies (Kock and Moqbel, 2021; An et al., 2022), technology efficacy positively predicted positive emotions and reduced anxiety but did not affect hopelessness or boredom. Enhancing social interaction (Qiu, 2022) and designing engaging curricula (Kwah et al., 2016) were highlighted as strategies to improve students' emotional experiences, emphasizing the role of instructional design. For achievement goals, outcome goals reduced boredom but were excluded from the positive emotion model, possibly due to the uncertainty of learning outcomes (Hu et al., 2024). Similarly, ability goals predicted pride and reduced hopelessness but were excluded from the negative emotion model, suggesting their limited link to acute emotional responses.

The results for gender and grade did not align with the hypotheses. Contrary to previous studies (Bender et al., 2012; Pekrun, 2017), which reported that girls experience higher levels of negative emotions than boys, this study found no significant gender differences in emotions. Descriptive statistics showed that girls scored slightly higher on positive emotions and lower on negative emotions than boys, potentially reflecting the influence of online learning environments. As Houlden and Veletsianos (2019) suggest, such environments provide greater flexibility and autonomy. This increased flexibility may help reduce traditional social pressures, thereby benefiting girls in particular. For grade, the results showed that it positively predicted all positive emotions and negatively predicted hopelessness and boredom but did not significantly influence anxiety. Although academic burden typically increases with grade level (Gao et al., 2022) and is often linked to negative emotions, a moderate level of burden may promote engagement and a sense of achievement. This balance might explain the observed positive association between grade and positive emotions.

In the following sections, we discussed the main predictors of online achievement emotions in more detail. Specifically, we examined the roles of the core predictors (control and value appraisals and negative effort belief), as well as grade level and school tier as important contextual factors.

### 5.1 Significant impact of control and value appraisals on achievement emotions

Control and value appraisals significantly influence achievement emotions in the online learning, positively predicting positive achievement emotions and inversely predicting negative achievement emotions. These findings align with the control-value theory framework, which posits that students' achievement emotions are determined by their perceptions of control over learning activities and the value they attribute to these tasks and outcomes (Pekrun, 2006). Prior studies in face-to-face contexts (Shao et al., 2020) and in online contexts for college students (Frenzel et al., 2007; Lichtenfeld et al., 2012; Noteborn et al., 2012) has similarly highlighted the important role of control and value appraisals in shaping achievement emotions. As Chen (2024) noted, students with high perceptions of control and value are more likely to experience positive achievement emotions, highlighting the importance of control and value appraisals regardless of the learning modality. The mechanisms underlying these findings can be well explained by control-value theory. Control appraisal enhances students' self-confidence and motivation (Duckworth et al., 2019), helping them engage more positively with learning challenges and reducing negative emotions. Similarly, value appraisal amplifies the perceived importance and meaningfulness of tasks (Goetz et al., 2014; Parker et al., 2021), fostering interest, focus, and enjoyment in learning, generating positive emotional experiences.

Interestingly, we found that while value appraisal was a stronger predictor for most emotions, control appraisal more strongly predicted pride. According to Hofmann and Fisher (2012), pride emerges from self-regulatory success, reinforcing commitment to future self-control by increasing the perceived importance of goals and enhancing conflict awareness when faced with temptations. This link to self-regulation may explain why control appraisal is a stronger predictor of pride.

Additionally, our mediation analyses showed that control and value appraisals not only directly predict achievement emotions but also mediate the relationship between technology efficacy and these emotions in online learning. This pattern is consistent with Stilin et al. (2024), who found that perceptions of competence and task value mediated the relationship between digital technology use and achievement emotions in science and mathematics learning. These findings highlight the important role of control and value appraisals as bridges linking external resources to students' emotional experiences, helping to explain how technology efficacy shapes these emotions.

### 5.2 Dual impact of negative effort belief on online achievement emotions

Negative effort belief had a significant positive influence on students' negative achievement emotions while negatively affecting their positive achievement emotions, consistent with previous findings in an online learning context with college students (Tempelaar et al., 2015). According to Weiner's (1986) attribution theory, casual attributions affect different emotions by shaping how individuals explain success and failure. Students who hold negative effort belief tend to attribute learning failures to uncontrollable factors, such as lack of ability, rather than lack of effort. These attributions weaken their sense of control (control appraisal decreases) and reduce their recognition of effort's value (value appraisal decreases). This psychological change exacerbates feelings of helplessness and frustration, which trigger negative achievement emotions.

Cultural context also shapes effort beliefs. In East Asian countries that believe in Confucian culture, diligence is commonly seen as the key to success, and sayings like “practice makes perfect” and “heaven rewards diligence” reinforce the belief that effort determines achievement (Wong and Wong, 2016). Some students persist in learning with this mindset, but others who see hard work fail to produce results, may begin to question the effectiveness of effort, which can contribute to the development of negative effort belief. At the same time, in competitive academic environments, repeated failures can erode confidence in effort's effectiveness (Wong and Wong, 2016), increasing stress and disengagement (Lin et al., 2020). Learned helplessness theory (Miller and Seligman, 1975) further supports this explanation by describing how repeated failures and misattributions to uncontrollable factors lead students to view effort as futile, which deepens their negative feelings of helplessness during the online learning process.

### 5.3 Grade and school tier differences in online achievement emotions

The results of regression analysis showed that grade was not a significant predictor of anxiety, but had significant, albeit small, effects on enjoyment, hopefulness, pride, hopelessness, and boredom. Although the regression coefficients were small, they suggest that grade-related differences in these emotions are statistically significant and warrant attention. Previous research indicates that school adaptation positively contributes to academic performance, such as improvements in mathematics achievement (Zhang et al., 2018). As students move into higher grades, they gradually adapt to the school environment, accumulate more learning experiences, and become more confident in managing academic tasks. This adaptation may explain why older students are more likely to experience positive emotions, such as enjoyment, hopefulness, and pride, while feeling less helpless or bored.

For school tier, the results revealed that students from higher-tier schools (e.g., key schools) reported higher levels of positive emotions, and lower levels of negative emotions. These findings align with prior research, which suggests that enriched learning environments foster a stronger academic self-concept and reduce emotional strain (Trautwein et al., 2006; Voisin et al., 2023). From the perspective of control-value theory, the superior resources, structured academic support, and effective teaching strategies in these schools likely help students build confidence and perceive greater value in their studies, fostering positive emotions. Moreover, these environments mitigate negative emotions by providing the tools and support needed to manage academic challenges effectively. Conversely, students in disadvantaged schools may face resource constraints and less personalized academic guidance, which can undermine their sense of control and value appraisals, exacerbating feelings of helplessness, boredom, and anxiety.

### 5.4 Implications

Several practical implications can be drawn from the study results regarding control appraisal, value appraisal, effort beliefs and technology efficacy. First, it is essential for elementary and middle school instructors to improve their instruction to facilitate students' sense of control over their learning. Teachers can improve instructional clarity by, for example, showing students the organizational structure of instructional content at the beginning of class. Even in schools with limited resources, teachers can achieve this by using simple tools like clear lesson outlines and downloadable lesson plans to afford a sense of learner control in online learning. Moreover, teachers should guide students in self-directed learning, especially as many lack online learning experience. This can be done through regular check-ins, peer discussions, or basic study planners.

Second, we recommend teachers and parents working together to help students recognize the value of learning because value appraisal plays a key role in both positive and negative achievement emotions. Teachers should link course content to real-life situations to demonstrate its relevance. For example, in mathematics, students can see how the subject contributes to academic success, career opportunities, and cognitive development. In resource-limited schools, leveraging local community resources can help achieve this goal. For instance, inviting community leaders, alumni and local professionals to share their life experiences can help students better appreciate the real-world relevance of their studies.

Third, measures should be taken to promote positive effort beliefs. Specifically, teachers and parents should help students understand the different outcomes of effort vs. its absence. They can share their personal stories or examples of success achieved through perseverance to demonstrate the value of hard work. Moreover, teaches also can encourage students to reflect on their achievements, which may strengthen their belief that effort leads to positive results.

Fourth, enhancing students' technology efficacy is important due to its impact on academic emotions. Schools can support this through regular training for both teachers and students, with tools that provide personalized, real-time feedback on their learning progress. For schools with limited resources, open-access platforms, such as China's national smart education platform, can help bridge the resource gap. Teachers can guide students in using these platforms through instructional videos or online meetings, thus helping them build confidence in using technology.

### 5.5 Limitations and future research

Several limitations should be considered when interpreting the findings of this study. First, achievement emotions are dynamic and may vary across situations and time. The use of a single self-report, as in this study, may not fully capture the variability of students' emotional experiences. Future studies could use longitudinal designs or real-time emotion tracking to better reflect these fluctuations. Second, although this study explored the mediating roles of control appraisal and value appraisal, it did not explore the causal relationships between these variables or explore how they interact to influence achievement emotions. Future research should consider structural equation modeling to uncover these mechanisms. Third, cultural context may limit the generalizability of the findings. This study focused on elementary and middle school students from China, where Confucian values emphasizing academic success and obedience to authority dominate. These cultural factors, along with high academic pressure, may influence how students experience and express achievement emotions. Therefore, the findings may not fully apply to populations in different cultural contexts. Future research should include more diverse cultural contexts to achieve greater generalizability of the results.

Despite these limitations, the key variables (e.g., control appraisal, value appraisal, and technology efficacy) explored in this study are general rather than context-specific, which suggests that the findings may still be generalizable to other contexts. Furthermore, the large sample size and geographically diverse selection of schools, including eight schools from Hubei province, Guangdong province, and Chongqing municipality, enhance the external validity of the findings. In sum, the findings of the current study both support and contradict with the existing findings of achievement emotions, providing new insights for understanding and designing online learning for elementary and middle school students.

## 6 Conclusions

Based on control value theory, this study integrated psychological, technological, and demographic factors to understand students' achievement emotions in online mathematics learning. From a psychological standpoint, control and value appraisals were the strongest predictors, enhancing enjoyment, hopefulness, and pride, while reducing anxiety, hopelessness, and boredom. Negative effort belief proved to be a powerful antecedent of negative emotions, even stronger than control appraisal. On the technological dimension, technology efficacy contributed to higher enjoyment, hopefulness, and pride, as well as lower anxiety. Importantly, mediation analysis showed that these effects were partially mediated by control and value appraisals, clarifying the mechanism by which technology efficacy influenced emotions. Finally, regarding demographic influences, grade level and school tier significantly affected emotional outcomes, with students in disadvantaged schools showing notably lower positive emotions and higher negative emotions, while gender did not show a significant effect in regression analysis. This study extended control-value theory to online mathematics education, offering practical implications for enhancing students' emotional experiences. It highlights the significance of integrating effort beliefs and technology efficacy into the framework, and provides guidance for improving emotional support in online learning, especially for students in disadvantaged school contexts.

## Data Availability

The datasets presented in this study can be found in online repositories. The names of the repository/repositories and accession number(s) can be found in the article/Supplementary material.
