# Supplementary material for: Predicting the achievement emotions of elementary and middle school students in online learning based on control-value theory
Source: Front Psychol. 2025 Jul 4;16:1601052. doi: 10.3389/fpsyg.2025.1601052 (PMC12272823; doi:10.3389/fpsyg.2025.1601052)
Supplement: Supplementary file 1 [file Supplementary_file_1.docx]

**Appendix A. The online learning experience questionnaire**

**Survey on Mathematics Learning During the "Suspending Classes Without Stopping Learning" Period**

Dear students,

Hello! Thank you for participating in this survey! In order to understand the online learning situation of students during the "Pause in Classes but Not in Learning" period for mathematics courses, we have some questions that require your response. Any information you provide will be used solely for academic research and will be kept strictly confidential. There are no right or wrong answers, so please fill in the information truthfully based on your actual situation. Thank you once again for your participation!

***The First Section***

**Basic Information**

1. Your gender is ___

A. Male B. Female

2. Your age is ___

3. Your location is ___

4. Your school is ___

5. Your grade is ___

***The Second Section (Five-Point Likert Scale)***

**Technology efficacy** **(seven items)**

6.I used to frequently participate in online learning.

7.I am proficient in using the current software for online learning on my own.

8.I am proficient in accessing various learning resources provided by the teacher (live videos, recorded videos, course materials, audio, assignments).

9.I am proficient in uploading assignments and reviewing feedback given by the teacher on my own.

10.I am proficient in using software to communicate and discuss with teachers or classmates.

11.I can skillfully express my thoughts and emotions in writing.

12.During online math classes, I am proficient in asking questions to the teacher or classmates.

**Value appraisal** **(five items)**

13.Performing well in online math classes is important to me.

14.Online math classes have provided me with practical knowledge.

15.I am interested in the content covered in online math classes.

16.Learning in online math classes is significant for me.

17.I can apply the knowledge gained in online math classes in many situations.

**Control appraisal** **(five items)**

18.I am confident that I can comprehend the most complex and difficult concepts in online math classes.

19.Considering the difficulty of online math classes, along with the teacher's guidance and my abilities, I believe I will perform well in class, assignments, and exams.

20.During online math classes, even when facing technical challenges, I can learn from the materials provided by the teacher.

21.Despite distractions, I can still excel in online math classes.

22.I believe I can learn mathematics even without the assistance of a teacher.

**Negative effort belief (two items)**

23.I believe that if I'm not intelligent, no matter how hard I study, I won't perform well.

24.If a subject is difficult for me, I am likely to struggle with it.

**Positive effort belief (three items)**

25.When something is challenging, I put in more effort to complete it.

26.The harder I work on something, the better I do.

27.The more challenging the task, the more I learn from it.

**Outcome goal (three items)**

28.Performing well in my studies is important to me.

29.I truly want to achieve good grades in exams.

30.One of my main goals while studying is to perform well.

**Ability goal (three items)**

31.It’s important for me to demonstrate my abilities through my assignments.

32.I want to prove my capabilities in school.

33.An important goal of mine is to prove my abilities through my learning.

***The Third Section (Five-Point Likert Scale)***

**The Achievement Emotions Questionnaire (AEQ)**

1. I enjoy being in the online math class.

2. I enjoy acquiring new knowledge in the online math class.

3. I am confident that I can do well in the online math class.

4. I have an optimistic view toward studying in the online math class.

5. I am proud of my performance in the online math class.

6. I’m proud of my ability to do well in the online math class.

7. I feel anxious when I think about going to the online math class.

8. Studying in the online math class makes me feel tense and uneasy.

9. I feel hopeless about having to attend the online math class.

10. I feel hopeless when I think about studying in the online math class.

11. I get bored in the online math class.

12. The study material in the online math class bores me to death.
