# Supplementary material for: Predicting the achievement emotions of elementary and middle school students in online learning based on control-value theory
Source: Front Psychol. 2025 Jul 4;16:1601052. doi: 10.3389/fpsyg.2025.1601052 (PMC12272823; doi:10.3389/fpsyg.2025.1601052)
Supplement: Supplementary file 2 [file Supplementary_file_2.docx]

**Appendix B. Correlation coefficients among key constructs**

|  | Grade | School tier | Technology efficacy | Control Appraisal | Value Appraisal | Outcome goal | Ability goal | Negative effort belief | Positive effort belief |  |
| --- | --- | --- | --- | --- | --- | --- | --- | --- | --- | --- |
| Grade | 1 |  |  |  |  |  |  |  |  |  |
| School tier | -.064** | 1 |  |  |  |  |  |  |  |  |
| Technology efficacy | | .041* | .214** | 1 |  |  |  |  |  |  |
| Control Appraisal | -.071** | .115** | .633** | 1 |  |  |  |  |  |  |
| Value Appraisal | -.060** | .121** | .681** | .701** | 1 |  |  |  |  |  |
| Outcome goal | -.046* | .104** | .480** | .448** | .573** | 1 |  |  |  |  |
| Ability goal | -.068** | .131** | .494** | .472** | .562** | .719** | 1 |  |  |  |
| Negative effort belief | 0.085** | -.108** | -.371** | -.380** | -.398** | -.281** | -.309** | 1 |  |  |
| Positive effort belief | -.155** | .075** | .476** | .531** | .573** | .490** | .544** | -.468** | 1 |  |

Note: **p* < 0.05, ***p* < 0.01, ****p* < 0.001.
