# Supplementary material for: Predicting the achievement emotions of elementary and middle school students in online learning based on control-value theory
Source: Front Psychol. 2025 Jul 4;16:1601052. doi: 10.3389/fpsyg.2025.1601052 (PMC12272823; doi:10.3389/fpsyg.2025.1601052)
Supplement: Supplementary file 3 [file Supplementary_file_3.docx]

**Appendix C. Summary of 12 single-mediator models examining the mediating role of control appraisal and value appraisal in the relationship between technology efficacy and achievement emotions.**

Figure 1 presents the single-mediator model used as the analytic framework for all mediation analyses conducted in this study.

**FIGURE 1** Mediation model

**
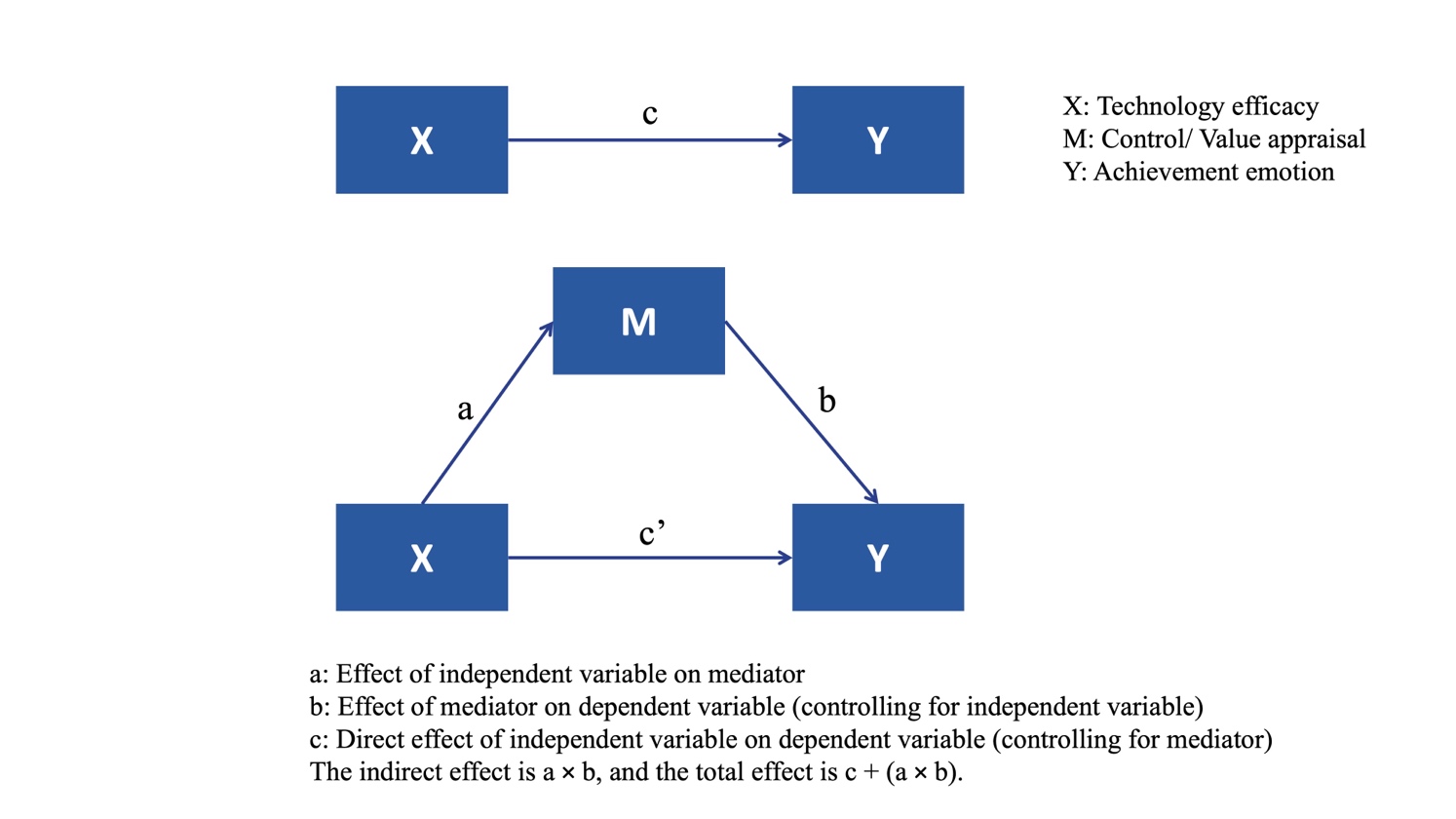
**

Equation 1 shows the total effect of technology efficacy on achievement emotions; Equation 2 shows the effect of technology efficacy on control/ value appraisal; Equation 3 shows the effect of both technology efficacy and control/ value appraisal on achievement emotions.

$\boldsymbol{Y}\boldsymbol{=cX+}\boldsymbol{e}_{\boldsymbol{1}}$ (1)

$\boldsymbol{M=aX+}\boldsymbol{e}_{\boldsymbol{2}}$ (2)

$\boldsymbol{Y}\boldsymbol{=c‘X+}\boldsymbol{b}\boldsymbol{M+}\boldsymbol{e}_{\boldsymbol{3}}$ (3)

Tables 1 to 12 summarize the results of 12 single-mediator models that test the effect of technology efficacy on six achievement emotions (enjoyment, hopefulness, pride, anxiety, hopelessness, and boredom), with either control appraisal or value appraisal as the mediator. Each table presents the standardized path coefficients (β), 95% confidence intervals (CI), and model fit indices (F, R²) for each model.

**TABLE 1 Mediation analysis for Technology Efficacy → Control Appraisal → Enjoyment.**

| Variable | Equation 1  (Enjoyment) | | Equation 2  (Control appraisal) | | Equation 3  (Enjoyment) | |
| --- | --- | --- | --- | --- | --- | --- |
|  | *β* | 95% CI | *β* | 95% CI | *β* | 95% CI |
| Technology efficacy | 0.600*** | [0.697, 0.768] | 0.631*** | [0.699, 0.764] | 0.290*** | [0.314, 0.394] |
| Control appraisal | — | — | — | — | 0.491*** | [0.483, 0.552] |
| F | 1652.175*** | | 1941.831*** | | 1499.345*** | |
| $R^{2}$ | 0.360 | | 0.398 | | 0.505 | |

*Note.* **p* < 0.05, ***p* < 0.01, ****p*< 0.001.

**TABLE 2 Mediation analysis for Technology Efficacy → Value Appraisal → Enjoyment**

| Variable | Equation 1  (Enjoyment) | | Equation 2  (Value appraisal) | | Equation 3  (Enjoyment) | |
| --- | --- | --- | --- | --- | --- | --- |
|  | *β* | 95% CI | *β* | 95% CI | *β* | 95% CI |
| Technology efficacy | 0.600*** | [0.697, 0.768] | 0.662*** | [0.609, 0.661] | 0.175*** | [0.176, 0.251] |
| Value appraisal | — | — | — | — | 0.642*** | [0.778, 0.856] |
| F | 1652.175*** | | 2293.891*** | | 2124.090*** | |
| $R^{2}$ | 0.360 | | 0.438 | | 0.591 | |

*Note.* **p* < 0.05, ***p* < 0.01, ****p*< 0.001.

**TABLE 3 Mediation analysis for Technology Efficacy → Control Appraisal → Hopefulness**

| Variable | Equation 1  (Hopefulness) | | Equation 2  (Control appraisal) | | Equation 3  (Hopefulness) | |
| --- | --- | --- | --- | --- | --- | --- |
|  | *β* | 95% CI | *β* | 95% CI | *β* | 95% CI |
| Technology efficacy | 0.638*** | [0.717, 0.782] | 0.631*** | [0.699, 0.764] | 0.287*** | [0.302, 0.372] |
| Control appraisal | — | — | — | — | 0.557*** | [0.534, 0.594] |
| F | 2019.774*** | | 1941.831*** | | 2148.555*** | |
| $R^{2}$ | 0.407 | | 0.398 | | 0.594 | |

*Note.* **p* < 0.05, ***p* < 0.01, ****p*< 0.001.

**TABLE 4 Mediation analysis for Technology Efficacy → Value Appraisal → Hopefulness**

| Variable | Equation 1  (Hopefulness) | | Equation 2  (Value appraisal) | | Equation 3  (Hopefulness) | |
| --- | --- | --- | --- | --- | --- | --- |
|  | *β* | 95% CI | *β* | 95% CI | *β* | 95% CI |
| Technology efficacy | 0.638*** | [0.717, 0.782] | 0.662*** | [0.609, 0.661] | 0.227*** | [0.232, 0.301] |
| Value appraisal | — | — | — | — | 0.621*** | [0.724, 0.797] |
| F | 2019.774*** | | 2293.891*** | | 2438.077*** | |
| $R^{2}$ | 0.407 | | 0.438 | | 0.624 | |

*Note.* **p* < 0.05, ***p* < 0.01, ****p*< 0.001.

**TABLE 5 Mediation analysis for Technology Efficacy → Control Appraisal → Pride**

| Variable | Equation 1  (Pride) | | Equation 2  (Control appraisal) | | Equation 3  (Pride) | |
| --- | --- | --- | --- | --- | --- | --- |
|  | *β* | 95% CI | *β* | 95% CI | *β* | 95% CI |
| Technology efficacy | 0.583*** | [0.711, 0.787] | 0.631*** | [0.699, 0.764] | 0.256*** | [0.287, 0.371] |
| Control appraisal | — | — | — | — | 0.518*** | [0.537, 0.610] |
| F | 1510.291*** | | 1941.831*** | | 1474.286*** | |
| $R^{2}$ | 0.340 | | 0.398 | | 0.501 | |

*Note.* **p* < 0.05, ***p* < 0.01, ****p*< 0.001.

**TABLE 6 Mediation analysis for Technology Efficacy → Value Appraisal → Pride**

| Variable | Equation 1  (Pride) | | Equation 2  (Value appraisal) | | Equation 3  (Pride) | |
| --- | --- | --- | --- | --- | --- | --- |
|  | *β* | 95% CI | *β* | 95% CI | *β* | 95% CI |
| Technology efficacy | 0.583*** | [0.711, 0.787] | 0.662*** | [0.609, 0.661] | 0.242*** | [0.267, 0.356] |
| Value appraisal | — | — | — | — | 0.514*** | [0.643, 0.735] |
| F | 1510.291*** | | 2293.891*** | | 1399.078*** | |
| $R^{2}$ | 0.340 | | 0.438 | | 0.488 | |

*Note.* **p* < 0.05, ***p* < 0.01, ****p*< 0.001.

**TABLE 7 Mediation analysis for Technology Efficacy → Control Appraisal → Anxiety**

| Variable | Equation 1  (Anxiety) | | Equation 2  (Control appraisal) | | Equation 3  (Anxiety) | |
| --- | --- | --- | --- | --- | --- | --- |
|  | *β* | 95% CI | *β* | 95% CI | *β* | 95% CI |
| Technology efficacy | -0.426*** | [-0.594, -0.509] | 0.631*** | [0.699, 0.764] | -0.230*** | [-0.351, -0.245] |
| Control appraisal | — | — | — | — | -0.310*** | [-0.393, -0.302] |
| F | 649.521*** | | 1941.831*** | | 461.365*** | |
| $R^{2}$ | 0.181 | | 0.398 | | 0.239 | |

*Note.* **p* < 0.05, ***p* < 0.01, ****p*< 0.001.

**TABLE 8 Mediation analysis for Technology Efficacy → Value Appraisal → Anxiety**

| Variable | Equation 1  (Anxiety) | | Equation 2  (Value appraisal) | | Equation 3  (Anxiety) | |
| --- | --- | --- | --- | --- | --- | --- |
|  | *β* | 95% CI | *β* | 95% CI | *β* | 95% CI |
| Technology efficacy | -0.426*** | [-0.594, -0.509] | 0.662*** | [0.609, 0.661] | -0.165*** | [-0.267, -0.160] |
| Value appraisal | — | — | — | — | -0.394*** | [-0.588, -0.477] |
| F | 649.521*** | | 2293.891*** | | 2124.090*** | |
| $R^{2}$ | 0.181 | | 0.438 | | 0.591 | |

*Note.* **p* < 0.05, ***p* < 0.01, ****p*< 0.001.

**TABLE 9 Mediation analysis for Technology Efficacy → Control Appraisal → Hopelessness**

| Variable | Equation 1  (Hopelessness) | | Equation 2  (Control appraisal) | | Equation 3  (Hopelessness) | |
| --- | --- | --- | --- | --- | --- | --- |
|  | *β* | 95% CI | *β* | 95% CI | *β* | 95% CI |
| Technology efficacy | -0.418*** | [-0.513, -0.438] | 0.631*** | [0.699, 0.764] | -0.210*** | [-0.285, -0.193] |
| Control appraisal | — | — | — | — | -0.329*** | [-0.363, -0.283] |
| F | 622.669*** | | 1941.831*** | | 464.155*** | |
| $R^{2}$ | 0.175 | | 0.398 | | 0.240 | |

*Note.* **p* < 0.05, ***p* < 0.01, ****p*< 0.001.

**TABLE 10 Mediation analysis for Technology Efficacy → Value Appraisal → Hopelessness**

| Variable | Equation 1  (Hopelessness) | | Equation 2  (Value appraisal) | | Equation 3  (Hopelessness) | |
| --- | --- | --- | --- | --- | --- | --- |
|  | *β* | 95% CI | *β* | 95% CI | *β* | 95% CI |
| Technology efficacy | -0.418*** | [-0.594, -0.509] | 0.662*** | [0.609, 0.661] | -0.110*** | [-0.171, -0.079] |
| Value appraisal | — | — | — | — | -0.465*** | [-0.599, -0.503] |
| F | 622.669*** | | 2293.891*** | | 618.320*** | |
| $R^{2}$ | 0.175 | | 0.438 | | 0.296 | |

*Note.* **p* < 0.05, ***p* < 0.01, ****p*< 0.001.

**TABLE 11 Mediation analysis for Technology Efficacy → Control Appraisal → Boredom**

| Variable | Equation 1  (Boredom) | | Equation 2  (Control appraisal) | | Equation 3  (Boredom) | |
| --- | --- | --- | --- | --- | --- | --- |
|  | *β* | 95% CI | *β* | 95% CI | *β* | 95% CI |
| Technology efficacy | -0.439*** | [-0.579, -0.500] | 0.631*** | [0.699, 0.764] | -0.212*** | [-0.310, -0.212] |
| Control appraisal | — | — | — | — | -0.359*** | [-0.423, -0.338] |
| F | 700.898*** | | 1941.831*** | | 543.788*** | |
| $R^{2}$ | 0.193 | | 0.398 | | 0.270 | |

*Note.* **p* < 0.05, ***p* < 0.01, ****p*< 0.001.

**TABLE 12 Mediation analysis for Technology Efficacy → Value Appraisal → Boredom**

| Variable | Equation 1  (Boredom) | | Equation 2  (Value appraisal) | | Equation 3  (Boredom) | |
| --- | --- | --- | --- | --- | --- | --- |
|  | *β* | 95% CI | *β* | 95% CI | *β* | 95% CI |
| Technology efficacy | -0.439*** | [-0.579, -0.500] | 0.662*** | [0.609, 0.661] | -0.092*** | [-0.161, -0.065] |
| Value appraisal | — | — | — | — | -0.525*** | [-0.722, -0.622] |
| F | 700.898*** | | 2293.891*** | | 780.841*** | |
| $R^{2}$ | 0.193 | | 0.438 | | 0.347 | |

*Note.* **p* < 0.05, ***p* < 0.01, ****p*< 0.001.
